# Drug–Drug Interaction Management Among Pharmacists in Jordan: A National Comparative Survey

**DOI:** 10.3390/pharmacy13050137

**Published:** 2025-09-28

**Authors:** Derar H. Abdel-Qader, Khalid Awad Al-Kubaisi, Esra’ Taybeh, Nadia Al Mazrouei, Rana Ibrahim, Abdullah Albassam

**Affiliations:** 1 Faculty of Pharmacy & Medical Sciences, University of Petra, Amman 11196, Jordan; 2 Department of Pharmacy Practice and Pharmacotherapeutics, College of Pharmacy, University of Sharjah, Sharjah 27272, United Arab Emirates; kalkubaissi@sharjah.ac.ae (K.A.A.-K.); nalmazrouei@sharjah.ac.ae (N.A.M.); ribrahim@sharjah.ac.ae (R.I.); 3 Department of Applied Pharmaceutical Sciences, School of Pharmacy, Isra University, Amman 11622, Jordan; esra.taybeh@iu.edu.jo; 4 Department of Pharmacy Practice, Faculty of Pharmacy, Kuwait University, Kuwait City 12037, Kuwait; albassam@hsc.edu.kw

**Keywords:** drug–drug interaction, hospital pharmacy, community pharmacy, patient safety, Jordan

## Abstract

**Introduction**: Drug–drug interactions (DDI) are a major, preventable cause of patient harm, a challenge amplified in Jordan by rising polypharmacy and documented high rates of medication errors. To date, no study in Jordan has systematically compared hospital and community pharmacists. This study aimed to conduct the first national, comparative assessment of DDI management among these two cadres. **Materials and Methods**: A national, cross-sectional study was conducted with 380 licensed pharmacists (175 hospitals, 205 community) recruited via proportionate stratified random sampling. A validated online questionnaire assessed demographics, objective DDI knowledge, professional attitudes, practices, and barriers. Multivariable logistic regression was used to identify independent predictors of high knowledge and optimal practice. All collected data were coded, cleaned, and analyzed using the Statistical Package for the Social Sciences (SPSS V28.0). **Results**: Hospital pharmacists achieved significantly higher mean objective knowledge scores than community pharmacists (10.3 vs. 8.1 out of 15, *p* < 0.001), a gap particularly wide for interactions involving high-risk OTC medications. The primary barrier for community pharmacists was a lack of access to patient data (85.4%), contrasting with high workload and physician resistance in hospitals. Optimal practice was independently predicted by higher knowledge (AOR = 1.25), a hospital practice setting (AOR = 3.65), and was inhibited by perceived physician resistance (AOR = 0.45). **Conclusions**: Jordanian hospital and community pharmacists operate in distinct worlds of knowledge and practice. A tailored, dual-pronged national strategy is essential. For hospitals, interventions should target interprofessional dynamics. For community pharmacies, health policy reform to provide access to integrated patient data is the most urgent priority. These findings highlight a globally relevant challenge of practice-setting disparities, offering a model for other nations to develop tailored, context-specific interventions to improve medication safety.

## 1. Introduction

Modern pharmacotherapy, while offering unprecedented therapeutic benefits, presents significant risks to patient safety, with adverse drug reactions (ADRs) being a leading cause of morbidity and hospitalisation globally [1]. A substantial and largely preventable portion of these ADRs stem from drug–drug interactions (DDI), a risk that is escalating in tandem with the rising tide of polypharmacy required to manage chronic diseases in aging populations [2]. This global challenge is particularly acute in Jordan, a nation undergoing a rapid healthcare evolution and facing its own growing burden of non-communicable diseases, which necessitates the concurrent use of multiple medications [3]. Recent local and regional research has revealed a startlingly high incidence of medication errors and pharmacovigilance challenges [4,5].

Pharmacists, as medication experts, are the cornerstone of medication safety and are uniquely positioned to identify, prevent, and manage DDIs [6]. Their role is critical in both hospital settings, where the documented impact of clinical pharmacists in drastically reducing prescribing errors by up to 76% underscores their value [7], and in community pharmacies, which serve as the public’s most accessible healthcare touchpoint [8]. The effectiveness of pharmacists in this vital function, however, is contingent not only on their clinical knowledge but also on their professional attitudes, daily practices, and the systemic environment in which they operate [9].

The operational ecosystems of hospital and community pharmacists, however, are fundamentally distinct. Hospital pharmacists are embedded within structured, multidisciplinary clinical teams, leveraging comprehensive patient data to manage complex, acute medication regimens [3]. In contrast, community pharmacists are on the frontline of primary care [8], facing the formidable challenge of making critical safety decisions amidst systemic pressures, such as high workload, physicians’ poor handwriting, and frequent interruptions [10,11], all of which are documented causes of dispensing errors in Jordan [12].

This dichotomy is crucial. The need for vigilant DDI management in the community setting is underscored by the expanding role of pharmacists in counseling on supplements and managing self-medication trends, which places an even greater onus on them to proactively identify risks [11,12]. Previous research in Jordan has shown that while educational interventions can reduce certain types of dispensing errors, they have a limited effect on these deep-seated, system-induced issues [13].

While regional and international studies have consistently identified knowledge gaps and suboptimal DDI management practices among pharmacists [2,14,15], these valuable studies have largely examined the profession as a monolithic entity. To our knowledge, no previous study in Jordan has systematically compared hospital versus community pharmacists regarding DDI management. This gap is not merely academic, it represents a critical blind spot in health policy, as an intervention focused on improving electronic prescribing (e-prescribing) skills for hospital physicians addresses a different root cause than an intervention designed to manage workflow interruptions in a busy community pharmacy, rendering a one-size-fits-all approach wholly inadequate [3]. Therefore, this study was conceived to assess the knowledge, attitudes, practices, barriers, and predictors of both high knowledge and optimal practice related to DDI management among licensed pharmacists in Jordan.

## 2. Materials and Methods

### 2.1. Study Design and Setting

This was a national, cross-sectional study conducted among licensed pharmacists in Jordan. The cross-sectional design was chosen to provide a comprehensive snapshot of the current state of DDI management across different practice settings within a defined timeframe. The study was carried out between January and March 2025.

### 2.2. Study Population and Sampling Strategy

The target population comprised all licensed pharmacists actively practising in either hospital or community pharmacy settings throughout Jordan. Pharmacists working in non-patient-facing roles (e.g., academia, pharmaceutical industry, regulatory affairs) and pharmacy students were excluded from the study.

A proportionate stratified random sampling technique was employed to ensure the sample was representative of the national pharmacist population. The official registry of the Jordan Pharmacists Association served as the sampling frame. The frame was first stratified by practice setting (hospital vs. community) and then by Jordan’s three main administrative regions (North, Central, and South). Participants were then randomly selected from each stratum using a computer-generated random number sequence.

### 2.3. Sample Size Calculation

The sample size was determined to ensure the findings would be statistically representative of the target population. According to data obtained from the Jordan Pharmacists Association, there were approximately 32,727 licensed pharmacists in Jordan at the time of the study [13].

Using the Raosoft online sample size calculator (Raosoft, Inc., Seattle, WA, USA), a minimum required sample size of 380 was established. This calculation was based on a 5% margin of error, a 95% confidence interval, and a 50% response distribution to ensure the most conservative sample size.

### 2.4. Data Collection Instrument and Validation

Data were collected using a structured, self-administered questionnaire, which was developed based on a comprehensive review of relevant studies in the field of DDI management [1,2,3,8,9] as well as the practical experience of the research team. The selected DDI pairs were chosen based on multi-faceted criteria: (1) high clinical significance (i.e., potential for severe harm), (2) high prevalence of use in Jordan, including common over-the-counter (OTC) medications, (3) inclusion of both pharmacokinetic and pharmacodynamic interactions, and (4) their use in previously validated international assessment tools to allow for comparability. The accuracy of the correct answers for the DDI scenarios was determined using the Lexicomp^®^ drug interactions database as the gold standard. While some interactions are mechanistically complex, a single-best-answer format was used, where the correct response reflected the highest level of clinical action required (e.g., ‘Contraindicated’ over ‘Monitor’). Also, the clinical relevance and prioritisation of the selected DDIs were contextualised for Jordan by reviewing the types of prescribing and dispensing errors documented in local studies [5,13].

The instrument was designed to be comprehensive and was divided into five distinct sections (Appendix A) as follows:

- Section A: Demographic and Professional Information: Collected data on age, gender, highest pharmacy degree, years of practice, geographical location, primary practice setting, number of daily prescriptions, continuous professional development, and daily workload.
- Section B: Knowledge of DDIs: Assessed both self-perceived competence and objective knowledge using a series of 15 validated drug-pair scenarios covering common and clinically significant DDIs.
- Section C: Attitudes towards DDI Management: Utilised a 5-point Likert scale to measure pharmacists’ professional beliefs and attitudes regarding their role in DDI screening.
- Section D: Practices in DDI Management: Quantified the frequency of key professional behaviours, such as screening prescriptions, counseling patients, and contacting prescribers.
- Section E: Resources and Barriers: Identified the primary information resources used by pharmacists and ranked the significance of potential barriers to effective DDI management.

The questionnaire underwent a rigorous validation process. Content validity was established by a multidisciplinary panel of five experts (two senior hospital pharmacists, two senior community pharmacists, and one senior academic pharmacist). A pilot test was then conducted with 20 pharmacists (not included in the final analysis) to assess clarity and comprehension. Internal consistency was calculated using Cronbach’s alpha, where all sections scored > 0.75, indicating high reliability of the questionnaire. The instrument was administered in English, the primary language of instruction in Jordanian pharmacy schools, thus not requiring translation into Arabic.

### 2.5. Outcome Measures and Definitions

- Self-Assessed DDI Knowledge: This qualitative outcome was measured using the single question in Section B Part 1: “How do you rate your own knowledge regarding drug–drug interactions?” Participants selected one of three options: ‘Poor/Fair’, ‘Good’, or ‘Very Good/Excellent’. For descriptive reporting, the ‘Good’ and ‘Very Good/Excellent’ categories were combined to represent a positive self-assessment of DDI knowledge.
- Objective DDI Knowledge Score: This quantitative outcome was calculated from the 15-item objective knowledge assessment. Each correct answer was awarded one point, creating a total score ranging from 0 to 15. This score was used as a continuous variable for descriptive statistics and for comparing means between groups. For the multivariable logistic regression, the score was dichotomised to identify pharmacists with a high level of knowledge, where a score of 12 or more (≥80% correct) was defined as ‘High Knowledge’.
- Perceived Workload: This was assessed via the single question, “How would you rate your daily workload?” Participants chose one of three response categories: ‘Low/Very Low’, ‘Moderate’, or ‘High/Very High’. For regression analysis, this was dichotomised, with ‘High/Very High’ representing a high perceived workload.
- Barrier Ranking: Barriers were ranked based on the percentage of respondents who rated them as either a “Moderate” or “Significant” barrier to their practice.

### 2.6. Data Collection Procedure

An online version of the questionnaire was created using Google Forms. An official invitation containing the study objectives, a consent statement, and a secure link to the survey was distributed to 600 randomly selected pharmacists through the official email and communication channels of the Jordan Pharmacists Association (e.g., SMS and WhatsApp messages). A total of 380 complete responses were received, for a response rate of 63.3%. The online form was configured to accept only one response per participant to prevent duplicate entries. Data collection was active for a period of 12 weeks, with two reminder emails sent to non-respondents at four-week intervals.

### 2.7. Ethical Considerations

The study protocol received full ethical approval from the Institutional Review Board (IRB) of the University of Petra (IRB Reference Number: O/1/2025). The first page of the online survey contained a detailed informed consent statement. Participants were required to actively consent by clicking “I agree to participate” before accessing the questionnaire. All data were collected anonymously and stored on a secure, password-protected server to ensure confidentiality.

### 2.8. Statistical Analysis

All collected data were coded, cleaned, and analysed using the Statistical Package for the Social Sciences (SPSS), Version 28.0 (IBM Corp., Armonk, NY, USA). The Shapiro–Wilk test was used to assess the normality of continuous data. As the data were found to be normally distributed (*p* > 0.05), parametric tests were employed.

Descriptive statistics (frequencies, percentages, means ± standard deviation [SD]) were used to summarise demographic data and all outcome measures. Inferential statistics, including Chi-square tests for categorical variables and independent *t*-tests for continuous variables, were used to assess associations between pharmacist groups and the main outcomes.

To identify independent predictors of high DDI knowledge (score ≥ 12/15), a multivariable logistic regression model was built. A two-step process was used: first, univariable logistic regression was performed for each potential predictor. Variables with a *p*-value < 0.25 in the univariable analysis were then included as candidates in the final multivariable model. Prior to the final analysis, multicollinearity among the independent variables in the model was assessed using Variance Inflation Factors (VIFs), no significant multicollinearity was detected (all VIFs < 5), indicating that the regression estimates are stable and reliable. For all final statistical tests, a *p*-value of <0.05 was considered statistically significant.

## 3. Results

### 3.1. Participant Demographics and Professional Characteristics

A total of 380 pharmacists were included in the analysis, composed of 175 (46.1%) hospital pharmacists and 205 (53.9%) community pharmacists.

The demographic and professional characteristics of the respondents are presented in Table 1. The mean age of participants was 34.2 ± 8.9 years, and a majority were female (80.0%). Statistically significant differences were found in the educational background and professional activities of the two groups. A higher proportion of hospital pharmacists held a Doctor of Pharmacy (Pharm.D) or postgraduate degree (41.7%) compared to community pharmacists (18.1%) (*p* < 0.001).

Furthermore, a significant difference was observed in continuing professional development (CPD) engagement (*p* < 0.001). Hospital pharmacists reported more frequent participation in clinical practice courses annually, with nearly half (49.7%) attending five or more courses per year, compared to just 21.0% of community pharmacists. Community pharmacists reported managing a significantly higher volume of prescriptions and perceived their workload to be higher than their hospital-based counterparts (*p* < 0.01).

### 3.2. Knowledge Regarding Drug–Drug Interactions

While 72.4% of all pharmacists self-assessed their DDI knowledge as “Good” or “Very Good/Excellent,” objective testing revealed significant knowledge gaps. The mean score on the 15-item objective knowledge assessment was 9.1 ± 3.2. Hospital pharmacists achieved a statistically significant higher mean score (10.3 ± 2.4) than their community-based counterparts (8.1 ± 3.5) (*p* < 0.001).

The detailed results in Table 2 show that while knowledge of life-threatening DDIs, such as sildenafil + isosorbide mononitrate was high, there were critical deficiencies elsewhere. For example, less than half of community pharmacists (49.3%) correctly identified the severe bleeding risk from combining warfarin + ibuprofen, a common OTC analgesic. Similarly, knowledge regarding the interaction between cyclosporine + clarithromycin, critical for transplant patients, was significantly lower among community pharmacists (40.5%) compared to hospital pharmacists (75.4%), highlighting a potential safety gap for patients transitioning care.

### 3.3. Attitudes Towards DDI Management

While pharmacists in both settings shared a strong positive attitude regarding their core professional duties, significant differences emerged in their confidence, inter-professional perceptions, and views on patient counseling (Table 3). A high percentage of both hospital (98.3%) and community (96.6%) pharmacists agreed that screening for DDIs is a fundamental responsibility. Similarly, both groups overwhelmingly endorsed the necessity of continuous professional development (CPD).

However, a significant gap was observed in professional confidence. Hospital pharmacists reported significantly higher confidence in both their clinical ability to identify DDIs (75.4% vs. 62.0%, *p* < 0.01) and their ability to communicate these concerns effectively to physicians (65.7% vs. 46.8%, *p* < 0.001).

Attitudes towards patient counseling also differed significantly. Community pharmacists were more likely to agree that providing DDI information often causes unnecessary patient anxiety (45.8% vs. 30.8%, *p* < 0.01), perhaps reflecting the challenges of counseling in a time-constrained environment without full clinical context. Conversely, community pharmacists felt a stronger sense of duty to educate patients on DDIs for non-prescription products compared to their hospital counterparts (*p* < 0.05).

### 3.4. DDI Management Practices

Self-reported practices revealed significant gaps between pharmacists’ positive attitudes and their daily actions, with profound differences observed between hospital and community settings (Table 4).

The practice of taking a brief medication history for new patients was significantly more common among hospital pharmacists (65.7% reporting “Often” or “Always”) compared to community pharmacists (35.1%). While screening of new prescriptions was frequent in both settings, the subsequent actions differed markedly. Hospital pharmacists were far more likely to contact the prescriber (85.7%) and document the intervention (74.9%). In stark contrast, only 45.4% of community pharmacists reported consistent documentation, and 17.1% stated they never or rarely document DDI interventions at all.

A critical finding emerged regarding advanced patient care. The practice of following up with a patient after a DDI intervention was found to be a major area for improvement across the board, with less than 20% of all pharmacists reporting that they perform this action “Often” or “Always”. This indicates a significant gap in the continuity of care and patient safety monitoring, a finding that warrants further investigation and targeted intervention.

### 3.5. Resources and Perceived Barriers

Digital databases (e.g., Lexidrug^®^, Micromedex^®^) were the most utilised resource by hospital pharmacists (81.1%), whereas community pharmacists relied on a mix of digital and AI-based databases (55.1%) and free online resources, such as Drugs.com (65.4%).

The most significant barriers were distinctly different for each group (Table 5). The top-ranked barrier for community pharmacists was a lack of access to patient’s complete medical/laboratory records (85.4%). For hospital pharmacists, lack of time and high workload were the predominant barriers (80.6%), followed by resistance or negative feedback from physicians (62.3%).

### 3.6. Predictors of High DDI Knowledge

To identify the factors independently associated with a high level of DDI knowledge, a multivariable logistic regression analysis was performed. The selection of variables for the model was based on their significance in a preliminary univariable analysis, only predictors with a *p*-value < 0.25 were included as candidates in the final multivariable model. The results of both the univariable and multivariable analyses are presented in Table 6. Several variables, including gender, geographic region, daily prescription volume, and perceived workload, were not significantly associated with DDI knowledge in the initial univariable analysis (all *p* > 0.25) and were therefore excluded from the final multivariable model.

The remaining variables, practice setting, age, educational background, years of practice, and CPD engagement, were entered into the multivariable regression. In the final adjusted model, three factors remained as significant independent predictors of high DDI knowledge. The strongest predictor was practice setting. After adjusting for other variables, hospital pharmacists were nearly three times more likely to possess a high level of DDI knowledge compared to community pharmacists (AOR = 2.87, 95% CI: 1.85–4.45).

Educational background was also a powerful predictor. Pharmacists holding a Doctor of Pharmacy (Pharm.D) or a postgraduate degree were more than twice as likely to have high knowledge (AOR = 2.11, 95% CI: 1.29–3.46). Finally, engagement in continuous professional development (CPD) was significantly associated with better knowledge, with those attending five or more courses annually being 1.8 times more likely to achieve a high knowledge score (AOR = 1.80, 95% CI: 1.15–2.81).

Interestingly, while both age and years of practice showed a weak but statistically significant association in the univariable analysis, these effects were no longer significant predictors after controlling for the stronger effects of practice setting, education level, and CPD engagement.

### 3.7. Predictors of Optimal DDI Management Practice

To identify the factors independently associated with optimal professional practice, a multivariable logistic regression analysis was conducted. The dependent variable, “Optimal Practice,” was defined as the consistent documentation of DDI interventions (selecting “Always” for the relevant survey question), a robust indicator of best practice. Documentation is a cornerstone of safe medication practice, providing a legal record, ensuring continuity of care, and serving as a key indicator of a pharmacist’s commitment to professional accountability. Therefore, consistent documentation was chosen as a robust and objective proxy for optimal practice.

The selection of variables for the final model followed a pre-specified two-step process, with the results of both the univariable and multivariable analyses presented in Table 7.

In the initial univariable screening, five variables showed a preliminary association with optimal practice (*p* < 0.25): practice setting, objective knowledge score, highest pharmacy degree, annual CPD engagement, and the perceived barrier of resistance from physicians. Several other factors, including age, gender, years of practice, geographic region, daily prescription volume, and perceived workload, were not significantly associated with practice in the univariable analysis (all *p* > 0.25) and were therefore excluded from the final multivariable model.

In the final adjusted model, three factors remained as significant independent predictors. The objective knowledge score was a key driver: for every one-point increase in a pharmacist’s knowledge score, the odds of them always documenting their interventions increased by 25% (AOR = 1.25, 95% CI: 1.10–1.41). However, the practice setting was the most powerful predictor, hospital pharmacists were over three and a half times more likely to consistently document interventions compared to their community counterparts (AOR = 3.65, 95% CI: 2.21–6.03). Furthermore, the professional environment was a critical factor, as pharmacists who perceived resistance from physicians as a significant barrier were 55% less likely to adhere to this best practice (AOR = 0.45, 95% CI: 0.28–0.72).

## 4. Discussion

This study was conceived to address a critical gap in the literature by providing the first national, comparative assessment of DDI management among Jordan’s hospital and community pharmacists. Our findings reveal a significant paradox: while both groups demonstrate a commendably positive attitude towards their professional responsibilities, this is undermined by a profound knowledge-practice gap. The results clearly delineate two distinct professional worlds, with hospital and community pharmacists possessing different knowledge strengths and facing unique, setting-specific barriers that impede the translation of positive attitudes into optimal practice.

The overwhelmingly positive attitude regarding the professional responsibility for DDI screening is a major strength identified in our study. Over 96% of pharmacists in both groups affirmed that this is their fundamental duty, a finding that strongly aligns with research from Saudi Arabia [1], Egypt [2], and Lebanon [8]. This shared professional ethos and the rejection of the notion that DDI management is solely the physician’s responsibility indicate a mature professional identity among Jordanian pharmacists and a readiness to embrace expanded clinical roles, a key theme in a previous study [3].

However, this positive foundation is immediately fractured by disparities in professional confidence and objective knowledge. Hospital pharmacists reported markedly higher confidence in their clinical ability and their communication skills with prescribers, which was corroborated by their significantly higher scores on the objective knowledge assessment. This superior knowledge in the hospital setting is consistent with the literature and is likely a direct result of their immersion in a structured clinical environment, their higher proportion of advanced degrees, and their daily management of complex patient cases with access to integrated health records [3]. The overall knowledge deficits we identified, particularly among community pharmacists, echo findings from across the region [2,9,14]. Our analysis provided granular insight into this gap, revealing that community pharmacists are particularly vulnerable in their understanding of interactions between prescription drugs and common OTC products, such as the interaction between warfarin and ibuprofen, a critical safety concern given the rise in self-medication [12]. This disparity in knowledge and confidence directly translates into a chasm between attitude and practice. While hospital pharmacists more consistently translated their perceived duty into action by contacting prescribers and documenting interventions, community pharmacists lagged significantly in these post-detection actions. This “know-do” gap is explained by the distinctly different barriers and resources inherent to each setting. The top-ranked barrier for community pharmacists, an overwhelming lack of access to patients’ complete medical records, was a fundamental systemic failure. This informational void forced them to make high-stakes decisions in the dark, a situation that directly elevates the risk of the very dispensing errors extensively documented in Jordanian community pharmacies [12].

In stark contrast, the primary barriers for hospital pharmacists were rooted not in a lack of information, but in the complexities of the clinical workflow and interprofessional dynamics. Their top-ranked barriers, high workload, resistance from physicians, and “alert fatigue”, were emblematic of a mature but stressed clinical environment. The issue of prescriber resistance was particularly telling, aligning with the findings of Jarab et al. [3], who identified interprofessional friction as a significant impediment in Jordanian hospitals, and Makkaoui et al. [8] in Lebanon. Similarly, “alert fatigue” is a well-documented phenomenon where an overabundance of low-priority warnings from decision support systems can lead to the dismissal of genuinely critical alerts [10,11]. This suggests that for hospital pharmacists, the challenge is not accessing information, but rather integrating it effectively and efficiently within a complex, human-centric system.

The identification of independent predictors for high DDI knowledge provides a critical roadmap for targeted educational and policy interventions. Our analysis revealed that DDI competency in Jordan was not randomly distributed but was strongly associated with the pharmacist’s practice setting, level of education, and commitment to lifelong learning. These findings both align with and add important nuances to the existing regional literature [2,3,15].

The most powerful predictor identified in our study was the practice setting, with hospital pharmacists being significantly more knowledgeable than their community counterparts. This corroborates the findings of Jarab et al. [3], who described the structured, multidisciplinary environment of Jordanian hospitals as a key facilitator of advanced pharmaceutical care. Hospital pharmacists’ daily immersion in complex clinical cases and access to integrated health records provides a continuous, real-world learning environment that is difficult to replicate in the community setting. This finding is echoed in international literature, where structured clinical environments consistently correlate with higher performance in medication safety tasks compared to more fragmented primary care settings [16,17]. This finding, however, contrasts with a large-scale study in Egypt which found no significant difference between the two groups, suggesting that the structural and educational divide between hospital and community pharmacy may be more pronounced in Jordan [2].

Our results also provided strong quantitative evidence for the value of advanced education. The finding that pharmacists with a Pharm.D or postgraduate degree were more than twice as likely to possess high DDI knowledge directly supports the conclusions of Jarab et al. [3], who noted that improved, clinically focused academic curricula are fostering stronger skills. This demonstrates a tangible return on investment for the more clinically oriented Pharm.D programmes in Jordan.

Perhaps the most nuanced finding related to professional experience. While univariable analysis suggested that pharmacists with more years of practice had slightly lower knowledge, a finding that could be attributed to a lack of exposure to modern curricula, this effect disappeared in the multivariable model. This was a critical insight. It suggested that it was not the duration of experience that dictated DDI knowledge, but rather the quality and nature of that experience. The significance of CPD engagement in the final model reinforced this: pharmacists who were actively learning were more knowledgeable, regardless of how long they have been practising. This underscores the conclusions of multiple studies calling for enhanced and mandatory continuous education to combat knowledge attrition and ensure pharmacists remain current [1,9]. Ultimately, our predictor analysis painted a clear picture: DDI expertise in Jordan is actively cultivated through advanced education, a commitment to ongoing learning, and practice within a supportive, data-rich clinical environment.

Perhaps the most critical contribution of this study was the quantitative explanation of the “attitude-practice paradox.” While our results showed that Jordanian pharmacists were attitudinally primed for their role, their daily actions were ultimately dictated by a combination of their knowledge, professional environment, and perceived barriers. The identification of independent predictors for optimal practice, specifically the consistent documentation of interventions, provides the crucial missing link in the “know-do” gap and has profound implications for health policy.

The fact that the objective knowledge score was a significant predictor of practice is a powerful finding. It provided quantitative evidence that enhancing a pharmacist’s clinical understanding has a direct, measurable impact on their professional behaviour, a pharmacist who truly understood the risk was more likely to perform the necessary actions to mitigate it. This reinforces the importance of the CPD and advanced education initiatives highlighted in our earlier analysis and by others [1,3].

However, our analysis powerfully demonstrated that knowledge operates within the constraints of the practice setting, which emerged as the strongest predictor of documentation, even after adjusting for knowledge. The structured clinical governance, integrated electronic records, and established interprofessional workflows in Jordanian hospitals create an ecosystem that enables and demands best practice [3]. This finding corroborates research from Europe which emphasizes that systemic factors, such as integrated health records and established workflows, are critical enablers of best practice [16]. In stark contrast, the community setting, lacking this infrastructure, presented significant hurdles to translating knowledge into consistent action. This was further supported by the finding that the perception of interprofessional barriers directly inhibits practice. Pharmacists who perceived resistance from physicians were significantly less likely to document their interventions, suggesting a defensive posture where a pharmacist may intervene verbally to protect the patient but will avoid creating a formal record that could lead to conflict, a sentiment previously described in qualitative studies but now quantified by our model [8]. Interestingly, while advanced education (Pharm.D) and CPD engagement were significant predictors of practice in the univariable analysis, their direct effect was no longer statistically significant in the final multivariable model. This was a critical insight. It suggested that the pathway to better practice was sequential: advanced education and CPD build higher knowledge, and it is this enhanced knowledge, combined with a supportive practice environment, that ultimately drives a pharmacist’s actions. The influence of education is therefore mediated through knowledge, and its impact on practice is blunted if the pharmacist’s work environment does not provide the necessary tools and support [3,4,10].

This study possessed several notable strengths, including its national scope, robust proportionate stratified random sampling strategy, and its novel comparative design, which provided a much-needed granular analysis of the pharmacy profession in Jordan. Nonetheless, several limitations must be acknowledged to ensure a balanced interpretation of the findings.

First, the cross-sectional design of the study allowed for the identification of significant associations but precluded the establishment of causality. For instance, while our analysis showed that higher knowledge was associated with better practice, we cannot definitively state that the knowledge caused the practice, it is also possible that pharmacists who engaged in better practice were more motivated to seek out knowledge.

Second, and perhaps most significantly, the reliance on self-reported data for the practice and attitude sections introduced the potential for social desirability bias. It is a well-established phenomenon that professionals, when surveyed about their own behaviours, may report actions that align more closely with professional ideals than with their actual daily routine. Therefore, it is plausible that the reported frequencies of positive behaviours, such as how often pharmacists contacted prescribers or documented interventions, were an overestimation of true practice. This suggests that the “attitude-practice paradox” we identified may be even more pronounced in reality. While this bias cannot be eliminated, the consistency of these self-reported findings with the objective knowledge scores, where the same gaps and patterns emerged, strengthens the overall validity of our conclusions about the differences between the two groups.

Third, the study was subject to a potential sampling and non-response bias. Pharmacists who were more professionally engaged, more confident in their knowledge, or who had a greater interest in the topic of DDI management may have been more likely to complete the survey. This could mean that our sample represented a “best-case scenario” of the Jordanian pharmacist population. Consequently, the true knowledge-practice gaps and the prevalence of systemic barriers in the wider, non-participating population may be even more pronounced than our findings suggested. Furthermore, the objective knowledge assessment tool did not include any non-interacting drug pairs, which limits its ability to assess for potential response bias or the over-identification of interactions. Future studies should incorporate such ‘true negative’ scenarios for a more robust evaluation.

## 5. Conclusions

In conclusion, while Jordanian hospital and community pharmacists were united in their commitment to preventing DDI-related harm, they operated in two vastly different worlds of knowledge and practice. Our findings provide a clear mandate that a one-size-fits-all national strategy for improving DDI management will fail. DDI expertise was not arbitrary but was significantly predicted by practice setting, advanced educational attainment, and active engagement in continuing professional development.

Therefore, fortifying the medication safety net for all Jordanians requires a tailored, dual-pronged approach. For hospital pharmacists, interventions must focus on structured interprofessional communication training to mitigate prescriber resistance and the implementation of smarter clinical decision support systems to combat alert fatigue. For community pharmacists, the most urgent priority is health policy reform. Advocating for a secure, integrated health information system that provides access to essential patient data is paramount. For community pharmacists, the most urgent priority is health policy reform. We explicitly recommend that advocating for a secure, integrated health information system that provides community pharmacists with access to essential patient data be made a national priority. Without this foundational tool, even the most extensive educational programs will have a limited impact on preventing DDI-related harm in the primary care setting.

## Figures and Tables

**Table 1:** Demographic and Professional Characteristics of Study Participants (n = 380).

Characteristic	Hospital Pharmacists (n = 175)	Community Pharmacists (n = 205)	Total Sample (n = 380)	*p*-Value
**Age (years), Mean ± SD**	32.8 ± 7.6	35.4 ± 9.7	34.2 ± 8.9	0.062
**Gender, n (%)**				0.731
Female	140 (80.0)	164 (80.0)	304 (80.0)	
Male	35 (20.0)	41 (20.0)	76 (20.0)	
**Highest Pharmacy Degree, n (%)**				<0.001 *
Bachelor of Pharmacy	102 (58.3)	168 (81.9)	270 (71.1)	
Pharm.D/MSc/PhD	73 (41.7)	37 (18.1)	110 (28.9)	
**Geographic Region, n (%)**				0.815
North	40 (22.9)	48 (23.4)	88 (23.2)	
Central	105 (60.0)	121 (59.0)	226 (59.5)	
South	30 (17.1)	36 (17.6)	66 (17.3)	
**Years of Practice > 10 years, n (%)**	58 (33.1)	81 (39.5)	139 (36.6)	0.198
**Annual CPD Courses (Clinical Practice), n (%)**				<0.001 * (*p*-for-trend < 0.001)
<5 courses	88 (50.3)	162 (79.0)	250 (65.8)	
5–9 courses	65 (37.1)	35 (17.1)	100 (26.3)	
≥10 courses	22 (12.6)	8 (3.9)	30 (7.9)	
**Prescriptions/Day, n (%)**				<0.001 * (*p*-for-trend < 0.001)
<50	70 (40.0)	21 (10.2)	91 (23.9)	
50–99	53 (30.3)	51 (24.9)	104 (27.4)	
100–149	21 (12.0)	48 (23.4)	69 (18.2)	
≥150	31 (17.7)	85 (41.5)	116 (30.5)	
**Perceived Workload, n (%)**				<0.01 *
Low/Very Low	30 (17.1)	25 (12.2)	55 (14.5)	
Moderate	65 (37.1)	55 (26.8)	120 (31.6)	
High/Very High	80 (45.7)	125 (61.0)	205 (53.9)	

* Statistically significant at *p* < 0.05. SD: Standard Deviation, CPD: Continuous Professional Development.

**Table 2:** Percentage of Responses for the Objective DDI Knowledge Assessment (n = 380).

Drug–Drug Interaction Pair	Hospital Pharmacists (n = 175) (%)	Community Pharmacists (n = 205) (%)	Total Sample (n = 380) (%)	*p*-Value
Sildenafil + Isosorbide Mononitrate	**94.3**	**90.2**	**92.1**	**0.134**
Warfarin + Sulfamethoxazole/TMP	91.4	80.5	85.5	<0.01 *
Aspirin + Warfarin	88.6	76.1	81.8	<0.01 *
Simvastatin + Clarithromycin	**85.1**	**65.4**	**74.5**	<0.001 *
Codeine + Diazepam	84.6	64.9	73.9	<0.001 *
Digoxin + Amiodarone	81.7	58.0	68.9	<0.001 *
Ibuprofen + Lisinopril	76.0	60.0	67.4	<0.01 *
Cyclosporine + Clarithromycin	75.4	40.5	56.6	<0.001 *
Methotrexate + Ibuprofen	74.3	47.3	60.0	<0.001 *
Lithium + NSAIDs (e.g., Ibuprofen)	82.3	48.8	64.2	<0.001 *
Sertraline (SSRI) + Tramadol	72.6	52.7	61.8	<0.001 *
Warfarin + Ibuprofen	70.3	49.3	58.9	<0.001 *
Ciprofloxacin + Theophylline	68.6	45.4	56.1	<0.001 *
ACE Inhibitor + Spironolactone	65.7	41.0	52.4	<0.001 *
Clopidogrel + Omeprazole	55.4	35.1	44.5	<0.001 *

*p-*value reflects the Chi-square test for significance between pharmacist groups. * Statistically significant at *p* < 0.05. Bold indicates an absolutely contraindicated interaction with the potential for immediate, life-threatening harm.

**Table 3:** Attitudes Towards DDI Management by Pharmacist Group (n = 380) (Reporting “% Agree/Strongly Agree”).

Attitude Statement	Hospital Pharmacists (n = 175) n (%)	Community Pharmacists (n = 205) n (%)	*p*-Value
Screening for DDI is a fundamental professional responsibility for all pharmacists.	172 (98.3)	198 (96.6)	0.281
I feel confident in my clinical ability to identify and assess the risk of most DDI.	132 (75.4)	127 (62.0)	<0.01 *
I am confident in my ability to effectively communicate a DDI concern to a prescribing physician.	115 (65.7)	96 (46.8)	<0.001 *
The primary responsibility for DDI management lies with the prescribing physician, not the pharmacist.	18 (10.3)	26 (12.7)	0.450
It is the pharmacist’s duty to proactively educate patients about potential DDI, even for non-prescription products.	145 (82.9)	185 (90.2)	<0.05 *
Providing DDI information to patients often causes unnecessary anxiety and may lead to non-adherence.	54 (30.8)	94 (45.8)	<0.01 *
Integrating reliable DDI software into the pharmacy workflow is the most effective way to improve patient safety.	158 (90.3)	171 (83.4)	<0.05 *
Continuous professional development (CPD) on DDI management is essential for my practice.	169 (96.6)	195 (95.1)	0.443

*p*-value reflects the Chi-square test comparing the proportion of “Agree” or “Strongly Agree” responses between the two groups. * Statistically significant at *p* < 0.05.

**Table 4:** Frequency of DDI Management Practices by Pharmacist Group (n = 380) (Reporting “Often” or “Always”).

Practice	Hospital Pharmacists (n = 175) (%)	Community Pharmacists (n = 205) (%)	*p*-Value
How often do you attempt to take a brief medication history (including medications from other pharmacies/doctors) for new patients?	65.7	35.1	<0.001 *
How often do you screen new prescriptions for potential DDI before dispensing?	91.4	86.8	0.155
How often do you proactively ask patients about their use of over-the-counter (OTC) medications?	58.3	70.7	<0.05 *
How often do you proactively ask patients about their use of herbal products or dietary supplements?	50.3	61.0	<0.05 *
When a clinically significant DDI is detected, how often do you contact the prescriber to recommend a change?	85.7	68.8	<0.001 *
How often do you explain to the patient the specific signs/symptoms to monitor for when a potential DDI is managed?	75.4	55.1	<0.001 *
How often do you document the DDI and the intervention you performed in a patient’s record or dispensing system?	74.9	45.4	<0.001 *
How often do you follow up with a patient after a significant DDI intervention to assess the outcome?	19.4	12.2	0.068

*p*-value reflects the Chi-square test comparing the proportion of “Often” or “Always” responses between the two groups. * Statistically significant at *p* < 0.05.

**Table 5:** Top-Ranked Barriers to DDI Management (Rated as “Moderate” or “Significant”).

**Barrier**	**Hospital Pharmacists (n = 175)(%)**	**Community Pharmacists (n = 205) (%)**	***p*** **-Value**
Lack of access to patient’s complete medical/lab records	45.1	85.4	<0.001 *
Lack of time/High workload	80.6	75.1	0.218
Resistance or negative feedback from physicians	62.3	33.7	<0.001 *
Insufficient clinical training on DDI management	21.7	30.2	0.076
Lack of a private area for patient counseling	35.4	58.0	<0.001 *
“Alert fatigue” from frequent software warnings	55.4	Not Applicable	-

* Statistically significant at *p* < 0.05.

**Table 6:** Univariable and Multivariable Logistic Regression Analysis of Predictors for High DDI Knowledge.

Characteristic	Crude Odds Ratio (COR) [95% CI]	*p*-Value	Adjusted Odds Ratio (AOR) [95% CI]	*p*-Value
**Practice Setting**				
Community Pharmacy	1.00	-	1.00	-
Hospital Pharmacy	3.15 [2.10–4.72]	<0.001 *	2.87 [1.85–4.45]	<0.001 **
**Age (per year increase)**	0.98 [0.96–0.99]	0.041 *	0.99 [0.97–1.01]	0.350
** Gender **			-	-
Male	1.00	-	-	-
Female	1.18 [0.78–1.80]	0.431	-	-
**Highest Pharmacy Degree**				
Bachelor of Pharmacy	1.00	-	1.00	-
Pharm.D/MSc/PhD	2.45 [1.56–3.85]	<0.001 *	2.11 [1.29–3.46]	<0.01 **
**Geographic Region**				
Central	1.00	-	-	-
North	0.92 [0.58–1.46]	0.723	-	-
South	1.15 [0.67–1.97]	0.611	-	-
** Years of Practice **				
<5 years	1.00	-	1.00	-
5–10 years	0.85 [0.55–1.32]	0.478	0.91 [0.58–1.44]	0.689
>10 years	0.62 [0.39–0.98]	<0.05 *	0.75 [0.45–1.25]	0.267
**Annual CPD Courses**				
<5 courses	1.00	-	1.00	-
≥5 courses	2.21 [1.47–3.32]	<0.001 *	1.80 [1.15–2.81]	<0.05 **
**Prescriptions/Day**				
<50	1.00	-	-	-
50–149	0.85 [0.54–1.34]	0.488	-	-
≥150	0.77 [0.47–1.26]	0.295	-	-
**Perceived Workload**				
Low/Very Low	1.00	-	-	-
Moderate	1.12 [0.65–1.94]	0.680	-	-
High/Very High	0.95 [0.56–1.61]	0.847	-	-

* Statistically significant at *p* < 0.25. ** Statistically significant at *p* < 0.05. Note: An assessment for multicollinearity was conducted prior to the final analysis, all Variance Inflation Factors (VIFs) for the variables included in the multivariable model were <5, indicating no significant collinearity.

**Table 7:** Univariable and Multivariable Logistic Regression Analysis of Predictors for Optimal Practice (Always Documenting Interventions).

Characteristic	Crude Odds Ratio (COR) [95% CI]	*p*-Value	Adjusted Odds Ratio (AOR) [95% CI]	*p*-Value
**Practice Setting**				
Community Pharmacy	1.00	-	1.00	-
Hospital Pharmacy	4.10 [2.65–6.34]	<0.001 *	3.65 [2.21–6.03]	<0.001 **
**Objective Knowledge Score** (per 1-point increase)	1.38 [1.24–1.53]	<0.001 *	1.25 [1.10–1.41]	<0.001 **
**Barrier: Resistance from Physicians**				
Not a Significant Barrier	1.00	-	1.00	-
A Significant Barrier	0.38 [0.25–0.58]	<0.001 *	0.45 [0.28–0.72]	<0.01 **
**Age (per year increase)**	0.99 [0.97–1.01]	0.315	-	-
**Gender**			-	-
Male	1.00	-	-	-
Female	1.08 [0.71–1.64]	0.718	-	-
**Highest Pharmacy Degree**				
Bachelor of Pharmacy	1.00	-	1.00	-
Pharm.D/MSc/PhD	1.85 [1.18–2.90]	<0.01 *	1.35 [0.82–2.22]	0.238
**Geographic Region**				
Central	1.00	-	-	-
North	0.37 [0.12–1.06]	0.852	-	-
South	0.95 [0.78–1.57]	0.411	-	-
** Years of Practice **				
<5 years	1.00	-	-	-
5–10 years	0.95 [0.68-1.02]	0.478	-	-
>10 years	0.89 [0.59–0.98]	0.54	-	-
**Annual CPD Courses (Clinical)**				
<5 courses	1.00	-	1.00	-
≥5 courses	1.98 [1.32–2.97]	<0.01 *	1.41 [0.89–2.23]	0.141
**Prescriptions/Day**				
<50	1.00	-	-	-
50–149	0.92 [0.61–1.38]	0.680	-	-
≥150	0.49 [0.31–1.66]	0.595	-	-
**Perceived Workload**				
Low/Very Low	1.00	-	-	-
Moderate	0.59 [0.25–1.04]	0.910	-	-
High/Very High	1.12 [0.84–1.92]	0.487	-	-

* Statistically significant at *p* < 0.25. ** Statistically significant at *p* < 0.05. Note: An assessment for multicollinearity was conducted prior to the final analysis, all Variance Inflation Factors (VIFs) for the variables included in the multivariable model were <5, indicating no significant collinearity.

## Data Availability

The data presented in this study are available on request from the corresponding author due to participant privacy and ethical restrictions.

## Appendix A. Questionnaire

Dear Pharmacist,

You are invited to participate in a national research study aiming to assess the current state of drug-drug interaction (DDI) management among pharmacists in Jordan. Your professional insights are invaluable for identifying strengths and areas for improvement in patient safety.

This survey is anonymous and will take approximately 15–20 min to complete. All data will be kept confidential and used only for the purposes of this research. Your participation is completely voluntary. By proceeding with this questionnaire, you are providing your informed consent to participate.

Thank you for your contribution to advancing pharmacy practice in Jordan.

**Section A: Demographic and Professional Information**

1. **Age:** ________ years
2. **Gender:** ☐ Male ☐ Female ☐ Prefer not to say
3. **Highest Pharmacy Degree Obtained:**
☐ Bachelor of Pharmacy (B.Pharm)
☐ Doctor of Pharmacy (Pharm.D)
☐ Master’s Degree (MSc) or PhD
4. **Geographic Region**
☐ North
☐ Central
☐ South
5. **Years of Practice since Graduation:**
☐ <1 year
☐ 1–5 years
☐ 6–10 years
☐ 11–15 years
☐ >15 years
6. **Annual CPD Courses (Clinical Practice)**
☐ <5 courses
☐ 5–9 courses
☐ ≥10 courses
7. **Primary Practice Setting:**
☐ Hospital Pharmacy
☐ Community Pharmacy (Independent & Chain)
8. **Average Number of Prescriptions You Personally Dispense/Review Per Day:**
☐ <50
☐ 50–100
☐ 101–150
☐ 151–200
☐ >200
9. **How would you rate your daily workload?**
☐ Low/Very Low
☐ Moderate
☐ High/Very High

**Section B: Knowledge of Drug-Drug Interactions (DDI)**

**Part 1: Self-Assessed Knowledge**

1. **How do you rate your own knowledge regarding drug-drug interactions?**
☐ Poor/Fair
☐ Good
☐ Very Good/Excellent

**Part 2: Objective Knowledge Assessment**

**Drug Pair**	**No Significant Interaction**	**Use with Caution/Monitoring Required**	**Contraindicated (Should Not Be Used Together)**	**I Don’t Know**
Sildenafil + Isosorbide Mononitrate				
Warfarin + Sulfamethoxazole/TMP				
Aspirin + Warfarin				
Simvastatin + Clarithromycin				
Codeine + Diazepam				
Digoxin + Amiodarone				
Ibuprofen + Lisinopril				
Cyclosporine + Clarithromycin				
Methotrexate + Ibuprofen				
Lithium + NSAIDs (e.g., Ibuprofen)				
Sertraline (SSRI) + Tramadol				
Warfarin + Ibuprofen				
Ciprofloxacin + Theophylline				
ACE Inhibitor + Spironolactone				
Clopidogrel + Omeprazole				

**Section C: Attitudes Towards DDI Management**

**Statement**	**Strongly Disagree (1)**	**Disagree (2)**	**Neutral (3)**	**Agree (4)**	**Strongly Agree (5)**
Screening for DDI is a fundamental professional responsibility for all pharmacists.					
I feel confident in my clinical ability to identify and assess the risk of most DDI.					
I am confident in my ability to effectively communicate a DDI concern to a prescribing physician.					
The primary responsibility for DDI management lies with the prescribing physician, not the pharmacist.					
It is the pharmacist’s duty to proactively educate patients about potential DDI, even for non-prescription products.					
Providing DDI information to patients often causes unnecessary anxiety and may lead to non-adherence.					
Integrating reliable DDI software into the pharmacy workflow is the most effective way to improve patient safety.					
Continuous professional development (CPD) on DDI management is essential for my practice.					

**Section D: Practices in DDI Management **

**Practice**	**Never**	**Rarely**	**Sometimes**	**Often**	**Always**
How often do you attempt to take a brief medication history (including medications from other pharmacies/doctors) for new patients?					
How often do you screen new prescriptions for potential DDI before dispensing?					
How often do you proactively ask patients about their use of over-the-counter (OTC) medications?					
How often do you proactively ask patients about their use of herbal products or dietary supplements?					
When a clinically significant DDI is detected, how often do you contact the prescriber to recommend a change?					
How often do you explain to the patient the specific signs/symptoms to monitor for when a potential DDI is managed?					
How often do you document the DDI and the intervention you performed in a patient’s record or dispensing system?					
How often do you follow up with a patient after a significant DDI intervention to assess the outcome?					

**Section E: Resources and Barriers**

**Part 1: Information Resources**

1. **Which of the following resources do you primarily use to check for DDI? (Select all that apply)**
☐ Digital Database (e.g., Micromedex^®^, Lexidrug^®^)
☐ Online free resources (e.g., Drugs.com, Medscape)
☐ AI-driven tools (e.g., GPT-4, Gemini)
☐ Printed reference books (e.g., BNF, Drug Information Handbook)
☐ Consulting a pharmacist colleague
☐ Consulting a physician
☐ Drug package insert
☐ My own memory/prior knowledge
☐ Other (please specify): _________________

**Part 2: Barriers to DDI Management**

**Barrier**	**Not a Barrier**	**Minor Barrier**	**Moderate Barrier**	**Significant Barrier**
Lack of access to patient’s complete medical/lab records				
Lack of time/High workload				
Resistance or negative feedback from physicians				
Insufficient clinical training on DDI management				
Lack of a private area for patient counseling				
“Alert fatigue” from frequent software warnings				
Lack of access to patient’s complete medical/lab records				
Lack of time/High workload				
